# The Situation of Hazardous Materials Accidents during Road Transportation in China from 2013 to 2019

**DOI:** 10.3390/ijerph19159632

**Published:** 2022-08-05

**Authors:** Shengxue Zhu, Shiwen Zhang, Hong Lang, Chenming Jiang, Yingying Xing

**Affiliations:** 1 Jiangsu Key Laboratory of Traffic and Transportation Security, Huaiyin Institute of Technology, Huaian 223003, China; 2 China Eastern Technology Application Research and Development Center Co., Ltd., Shanghai 201700, China; 3 The Key Laboratory of Road and Traffic Engineering of Ministry of Education, College of Transportation Engineering, Tongji University, Shanghai 201804, China; 4 China Institute of FTZ Supply Chain, Shanghai Maritime University, Shanghai 201306, China

**Keywords:** hazardous materials accidents, road transportation, accident characteristics, historical analysis

## Abstract

The safety situation of hazardous materials (hazmat) accidents during road transportation in China is severe and very serious accidents occurred frequently. Such accidents not only have a huge impact on the environment but also have serious consequences for people and the economy, such as fires and explosions. Therefore, it is necessary to understand the characteristics and laws of road transport accidents of hazmat systematically. This paper investigated 2777 hazmat transportation accidents in China from 2013 to 2019 to identify the characteristics, consequences, and causes of the accident. The results show that August (10.05%) and December (9.76%) are the peak periods of hazmat transportation accidents, while most hazmat transportation accidents occurred in the early morning (6:00–9:00 a.m.) and at noon (9:00 a.m.–12:00 p.m.) hours. For the geographical location, the accidents mainly occurred in the east China (34.35%) and the northwest China areas (14.87%). The main types of hazmat transportation accidents were rollover (35.36%), rear-end (22.58%), and collision (14.87%), where the probability of a major leak was high. The most common hazmat transportation accidents involve gas (17.79%), flammable liquid (56.07%), and corrosive substance (12.28%). The most common consequences of the hazmat transportation accidents were leakage (80.34%), followed by fire release (8.32%) and explosion release (2.34%). Human factor (26.74%) is the main cause of hazmat transportation accidents. These findings could help hazmat transportation managers and planners develop appropriate measures for improving hazmat transportation safety.

## 1. Introduction

With the acceleration of the Chinese industrialization process, the production of hazardous materials and logistics increased sharply. On one hand, new developments have been injected into social and economic development. On the other hand, the safety of hazmat transport has become increasingly prominent. From the statistics of recent years, the national road accidents of hazmat transportation have gradually increased and pose a serious threat to people’s lives. In 2015, there were 419 hazmat transportation accidents, and 36.04% of these accidents resulted in serious crashes and injuries (State Administration of Work Safety, SAWS). Although the amount of hazmat accidents is decreasing due to effective management strategies of the Government administration unit in China, the serious consequence of crashes is increasing. Therefore, a systematic analysis of hazmat transportation accidents is needed to prevent the crashes from the source.

Historical surveys covering the characteristics and frequency of hazardous material transport accidents have been a very active area of research in the past few decades [1]. Traffic accidents involving hazmat are one of the main types of accidents affecting road transport safety [2], which caused huge economic losses directly and damage to personnel and the environment indirectly. Researchers and practitioners have made great efforts to improve the safety of hazmat. In addition, historical accident data were also used to identify the factors affecting the safety of hazmat transportation and to determine the impact of different factors on the safety of hazmat transportation, all of that is to find better ways to prevent or reduce the occurrence of accidents.

Vincenzo Torretta et al. [3] analyzed the risks of hazmat transportation, which are the substances being transported and the characteristics of the road network, such as the type of road, the weather conditions, the skills of the driver, and the concentration of the population along the selected route, and review the experience of using different decision support systems over the past few years. In the fault tree analysis of reliability research [4], human factors, vehicle factors, environmental factors, and freight factors are the main factors causing hazmat accidents. Through analysis, human factors account for a large proportion of these. Therefore, the evaluation of factors in this link should be strengthened. Tanackov et al. [5] used 9467 hazmat accident samples in the Failure and Accidents Technical information System (FACTS) database to study the impact of factors on hazmat transportation. According to the consequences and location of the accident, the reference risk in the logistics subsystem is calculated and showed a significant impact on risk allocation. Based on the comprehensive analysis of the road transportation network topology and hazmat risk characteristics during road transportation, an Impact Strength model was developed to assess hazmat road transportation vulnerability [6], which could reduce road transportation risk in advance and build an early road transportation risk warning mechanism. In addition, 1932 accidents in over 95 countries (1931–2004), 322 accidents in China (2000–2008), and 2046 accidents investigated in China (2013–2017) were selected and analyzed for verifying the methodology by a combination of normal distribution and an *f–N* curve [7]. It showed that fatal accidents fitting in mean value curves is even better than the linear curves by groups of transport modes, countries, and periods.

Moreover, the data on hazmat transportation have been used frequently by many researchers [8,9,10,11,12,13]. For example, the data of 4638 accidents in the road transportation of hazardous products were selected for exploring the evolution and the recent scenario of accidents [8]. It was concluded that high concentration of accidents was around 10:00 a.m., tipping over was the main cause of accidents, flammable liquids were the most frequent hazardous product, and so on. Warden et al. [9] also used the data of crashes occurring from 2000 to 2006 and concluded that hazmat-related crashes were associated more frequently with late evening/early morning hours and under artificial lights or at dusk other than different road surface conditions, weather conditions, or cause of the crash. Shen et al. [10] described 708 tanker accidents associated with hazmat transportation in China from 2004 to 2011 and analyzed the causes, location, types, time of occurrence, the hazardous class for materials involved, consequences, and the corresponding probability. The conclusion is that the occurrence time and hazmat class are the same consequence as previous literature, while the road surface and cause are also the factors related to crashes, which are opposite to Warden et al. [9]. Bunn et al. [11] identified and characterized transportation incident management-related occupational fatalities data from 2005 to 2016. It showed that law enforcement vehicle pursuit, towing and recovery vehicle loading, and disabled vehicle response were high-risk activities that led to transportation incident management (TIM)-related occupational fatalities. Besides, some other driver violations, such as hit-and-run crashes, can also delay the rescue of the crash victims and increase the potential injury severity [14,15]. As well, Hong et al. [16] focused on finding the key factors that have an impact on the accident using a statistical method based on crash data from 2007 to 2017. Cold weather, severe crashes and bigger trucks, leakage, and tunnel locations [17,18] can increase accident duration. Wang et al. [19] summarized the fire hazards in China during the period from 2000 to 2016 and concluded that the average GDP, the month (January, April, October, and December), the time of 10:00 to 22:00, residential buildings, and factory buildings possessed a high fire hazard potential. In addition, 653 representative cases of surface water pollution accidents in China were identified and described as a function of time, location, materials involved, origin, and causes by Yao et al. [20]. The conclusion is that the proportion of accidents originating from mini and small-sized chemical plants was high risk. Duan et al. [21] analyzed hazardous chemical accidents in China from 2000 to 2006. The accident rate in developed southeast coastal areas was far higher than that in the northwest regions. Nearly 80% of hazmat accidents occurred in small and medium-sized enterprises. Furthermore, a study of 322 hazmat accidents that occurred from 2000 to 2008 was carried out [22]. Release (84.5%) is the most frequent type of accident. Liu et al. conducted a statistical analysis of hazardous material accidents (HMAs) in China from 2013 to 2018, revealing different time volatility, spatial distribution, and accident consequence features [23]. However, they provided few discussions on the hazmat factors, such as hazmat class.

Above all, the aforementioned studies have provided useful findings on the characteristics and causes of hazmat transportation accidents. However, there have been few studies that conducted a systematic analysis of the trends of hazmat accidents during road transportation in China in recent years. Motivated by this observation, this paper analyzes the historical data of hazmat transportation, such as the time, space, accident type, road type, consequences, and causes of hazmat accidents during road transportation, to obtain the basic characteristics and laws of the accident, which will provide support for accident prevention and security technology related to hazmat transportation.

## 2. Data Source and Methodology

The hazmat accident data used in the paper were collected from the State Administration of Work Safety (SAWS), which takes charge of the collection and analysis of all industrial production accidents that occur in China and regularly releases these data to the public [24]. To improve chemical safety and prevent accidents, the National Registration Center for Chemicals (NRCC) of SAWS has developed a petrochemical accident analysis and a data interpretation platform for hazmat accidents statistics, analysis, and interpretation. In addition, the China Chemical Safety Association (CCSA), as an affiliation of SAWS, owns and manages a chemical-safety-related database: The Chemical Accident Cases. Combining these two databases, detailed information on chemical accidents throughout China could be obtained, including numerical (number of injuries and deaths) and qualitative information (substances involved, type of process). It should be noted that the data for Taiwan, Hong Kong, and Macao were not obtained in this study.

The *f–N* curve is a method expressing the results of risk evaluation, which is usually used in the hazmat factors risk assessment [7,22]. It has been used to analyze accident fatality statistics based on the accumulated frequency–number of deaths graphs in many studies [25,26,27]. In most cases, it refers to the frequency of a certain number of casualties and a means of indicating the results of a risk analysis. The mathematical expression of *f-N* is:

(1) $$P_{\left(x\geq N\right)}=F_{j}=\frac{\sum_{i=j}^{n}N_{i}}{\sum_{i=1}^{n}N_{i}}$$

where *N* is the number of deaths (*x*-axis); $P_{\left(x\geq N\right)}=F_{j}$ is the probability that the number of deaths is equal to or more than N (*y*-axis) in an accident; N is the total number of categories or rankings, and $N_{i}$ is the number of accidents in a given category i.

To assess the severity of fatal accidents in the transport of hazmat, the most common method is to plot the *f–N* curve by showing the relationship between cumulative frequency and the number of deaths. So, hazmat transportation accidents with at least one person’s death, serious injuries, and minor injuries were selected and grouped according to the number of deaths, serious injuries, and minor injuries in this paper.

This paper focused on the hazmat accidents during road transport, and accidents that occurred during loading, unloading, storage, and maintenance were excluded. As a result, 2777 hazmat transportation accidents in China during 2013–2019 were derived from the database. Each record covers the date, time, location, vehicle type, the number of vehicles involved, the quantity and categories of hazmat, accident type, main causes, deaths and casualties, and a detailed description of the accident. Thus, risk factors considered in this study were classified into hazmat factors, driver factors, location factors, environment factors, vehicle factors, accident factors, and consequences.

## 3. Results and Discussion

### 3.1. Distribution of Accidents by Time

**Figure 1:**
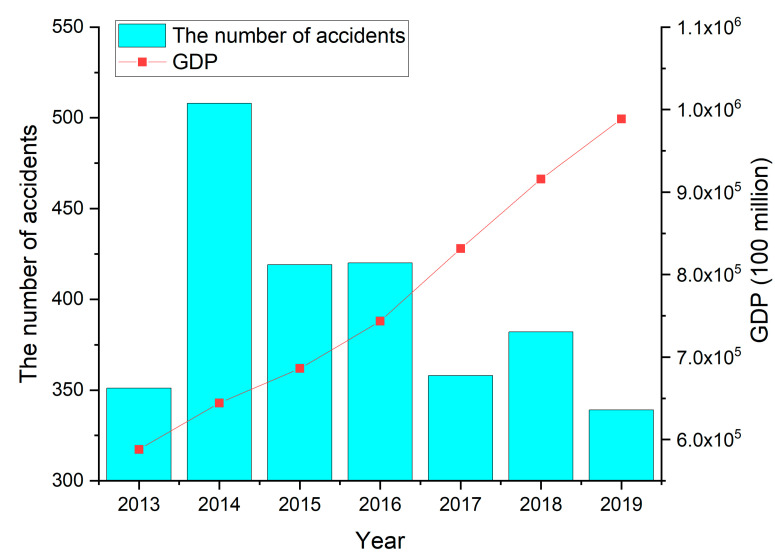
Distribution of accidents from 2013 to 2019.

The year of hazmat accidents distribution is plotted in Figure 1, from which it can be seen the number of accidents has been declining year by year although it increases by about 157 accidents from 2013 to 2014. The decrease in the number of accidents from 2016 to 2017 is larger than that of other years. This is mainly because hazmat transport vehicles on the most domestic highways are prohibited at specific times on specific road sections. From 2013 to 2019, the overall number of accidents declined, but it increased significantly in 2014 (Figure 1). In addition, the road transport of hazmat has a relatively increasing trend with the increase in GDP, which explains the severe situation of hazmat transportation accidents to some extent. The number of accidents in 2015 dropped sharply, and the number of accidents continued to rise slightly in the following years. According to statistics, from 2013 to 2019, the number of vehicles carrying hazmat on the road increased year by year, but the number of corresponding accidents has eased, indicating that safety measures have been taken and relatively successful. However, the severity of the accident has increased. Therefore, the government should take various measures to prevent and control such accidents.

**Figure 2:**
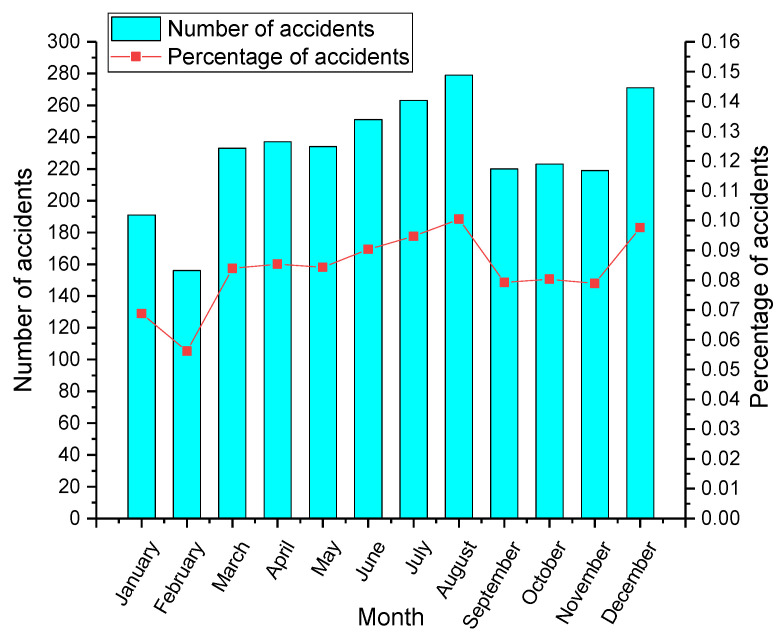
Distribution of accidents by months.

Figure 2 is the monthly distribution of hazmat accidents from 2013 to 2019. It reveals that August is the month with the highest accident probability, followed by December (10.05% and 9.76%, respectively). August is the month when the major accidents are relatively concentrated. High temperature, high humidity, rain, typhoons, and floods in summer have obvious impacts on safe transportation, increasing the risks of fire, explosion, and leakage of hazmat transportation. In addition, the weather in December always changes. The climate is cold, and there is more rain and snow, so the accident rate is also higher. Comparatively speaking, there is a lower proportion of accidents between January and February. This may be attributed to the Spring Festival, during which few traffic vehicles with hazardous materials began travelling. As a result, hazmat transportation accidents will naturally decrease. This result is also consistent with the previous study [23].

**Figure 3:**
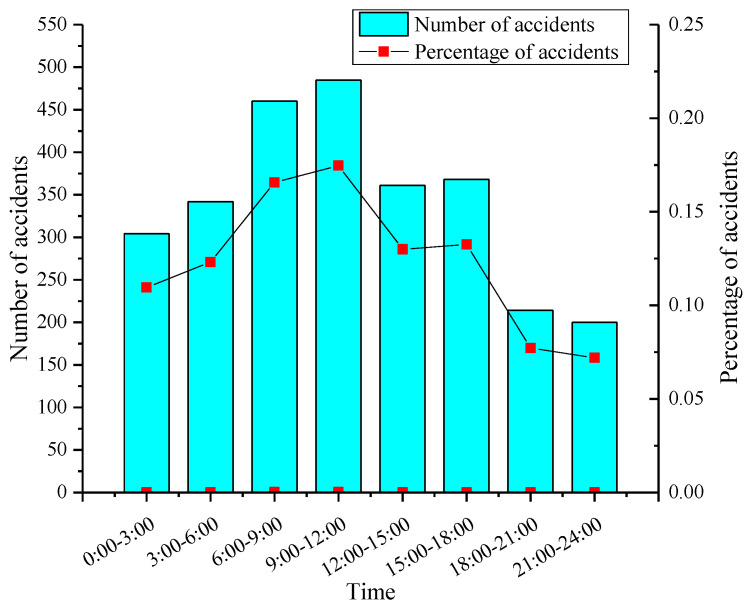
Distribution of accidents by time of day.

For the case of 2777 samples with more accurate time, relevant statistics were found. Hazmat transportation accidents are more likely to occur from 6:00 to 9:00 a.m. and 9:00 to 12:00 a.m., while there is a significant downward trend of accidents from 18:00 to 24:00 p.m., as shown in Figure 3. This is mainly due to lower traffic volume during nighttime. However, as time goes by, the number of accidents gradually increase. The lower visibility for driving and the fatigue of drivers in the early morning contribute to an increase in the number of accidents from 0:00 to 6:00 a.m. Additionally, the maximum number of hazmat transportation accidents happened from 9 to 12 a.m. The most possible reason is that most of hazmat transport vehicles begins travelling and enters highways from 6:00 to 9:00 a.m., resulting in the maximum truck rate of traffic flow during the period 9 to 12 a.m. This result is in line with the previous study [10].

### 3.2. Distribution of Accidents by District and Province

**Figure 4:**
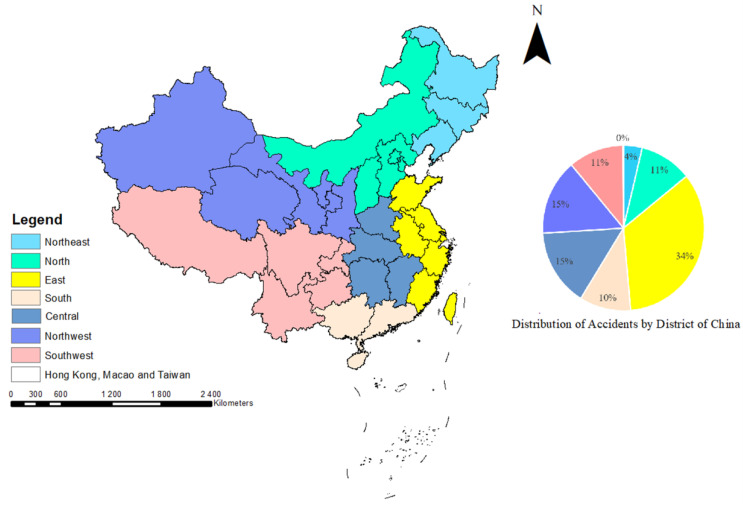
Division of different regions of China and distribution of accidents by district.

**Figure 5:**
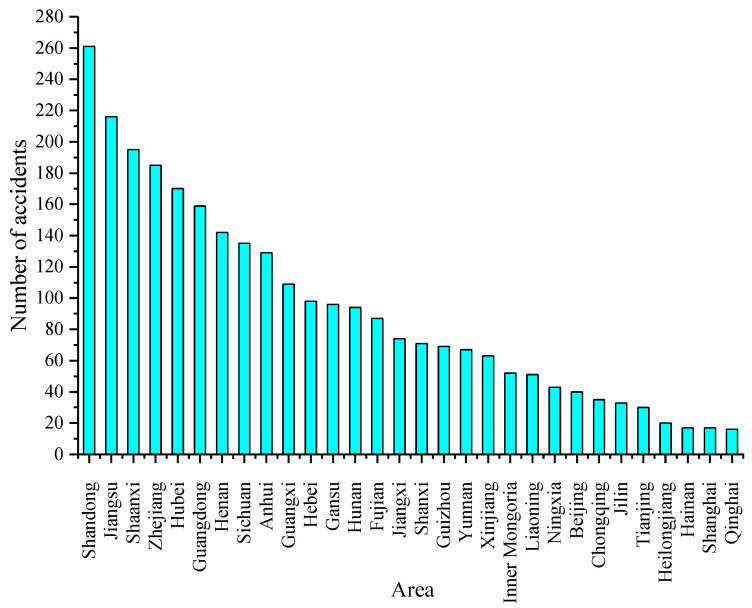
Distribution of accidents by the province of China.

**Figure 6:**
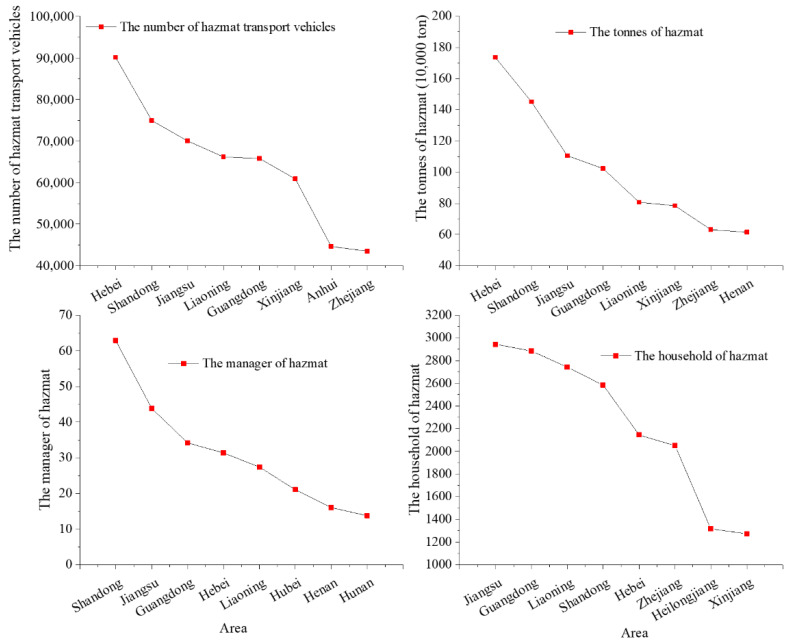
Distribution of hazmat transport vehicles and tons and the number of hazmat managers and households by the province of China.

According to geographical and economic features [28,29], different provinces of China are divided into several regions: Northeast, North, East, South, Central, Northwest, and Southwest China (Figure 4). Figure 4 shows that the number of hazmat transportation accidents in East China is the highest (accounting for 34.35%). This is mainly because three of top four provinces and cities with hazmat transportation accidents are located in East China. As shown in Figure 5, there were 261 hazmat transportation accidents in Shandong, ranking in the top, followed by Jiangsu, Shaaxi, and Zhejiang Provinces, which had 216, 195, and 185 hazmat transportation accidents, respectively. The most probable reason is that the road transportation of hazmat is mostly short-distance transport. Therefore, the location of accidents during transportation was positively correlated with the location of manufacturers and carriers of hazmat. Figure 6 shows the distribution of hazmat transport vehicles and tons, and the number of hazmat managers and households by the province of China. Consistent with the number of accidents, Shandong (9.4%), Jiangsu (7.8%), and Zhejiang (6.7%) provinces also had the large number of hazmat transport vehicles, tons, managers (drivers, escorts, and loaders), and households. This reveals that the prosperity of the hazmat transportation would naturally increase the probability of hazmat transportation accidents. Meanwhile, no accidents occurred in Tibet owing to the smaller scales of industries and hazmat traffic existing in these areas.

### 3.3. Distribution of Casualties by Road Class and Cause

The populations affected by the hazmat transportation accidents were expressed according to the scale of the consequences: number of slightly injured, number of seriously injured, and number of deaths.

#### 3.3.1. Number of People Slightly Injured

The number of people slightly injured accounts for 5.97% of the total number of accidents. Of those accidents that did cause slight injuries, a high percentage (62.7%) involved one person slightly injured, 32.5% involved two to five people slightly injured, and only eight involved more than five people slightly injured.

**Figure 7:**
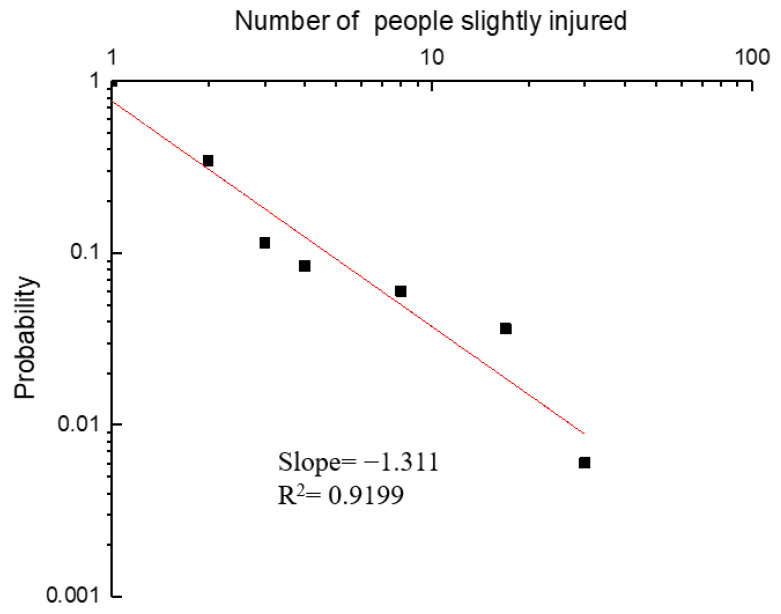
Accumulated probability of an accident with *N* slightly injured.

Equation (1) was used to calculate the cumulative probability or frequency of the number of people slightly injured. For the number of slightly injured 1<N<30, the function type fitted by the minimum square method in the form of $P=N^{b}$, and b=−1.311 (Figure 7). A straight line with a slope of −1.311 was obtained by plotting the data on a log–log axis system. Figure 7 can be used to estimate the cumulative probability relationship between different slightly injured. For example, the probability of a slightly injured involving 10 or more than 10 is 4.22 times greater than that of a slightly injured involving 30 or more.

#### 3.3.2. Number of People Seriously Injured

**Figure 8:**
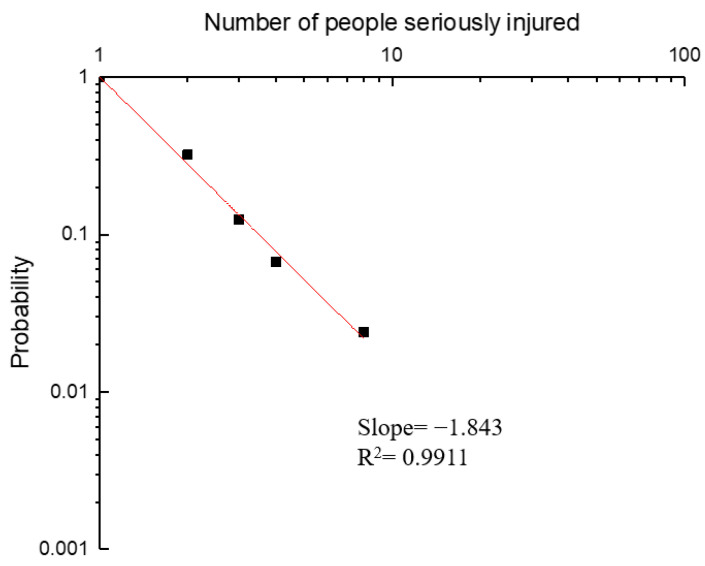
Accumulated probability of an accident with *N* seriously injured.

As in the previous section, the number of people seriously injured (209) was also grouped into several categories (1, 2–5, and over 5). Hazmat transportation accidents resulting in one person seriously injured accounted for 66.0% of the total number of accidents. In 88.7% of the remaining cases, between two and five people were seriously injured. Only eight accidents caused more than six serious injuries. The cumulative probability of the number of serious injuries was derived using Equation (1). The results are shown in Figure 8. For 1<N<10, the best fit (minimum square method) for a curve of type is $P=N^{b}$ and gave b=−1.843 A straight line with a slope of −1.843 was obtained by plotting the data on a log–log axis system.

#### 3.3.3. Number of Deaths

Most hazmat transportation accidents (>92.0%) are not considered fatal, with the total number of deaths reaching 564. Of the 222 fatal accidents, a high percentage (60.0%) involved 1 death, and 35.6% involved 2–5 deaths. Additionally, the maximum number of deaths caused by hazmat transportation accident could be as high as 43 deaths, indicating the serious consequences of hazmat transportation accidents.

**Figure 9:**
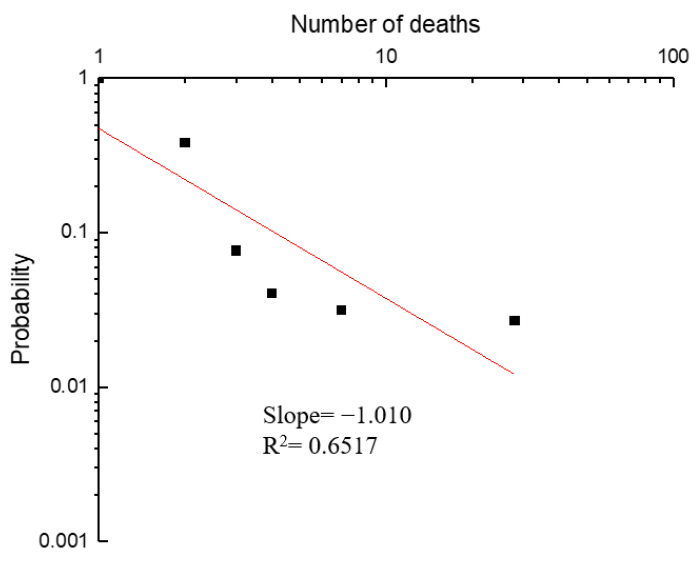
Accumulated probability of an accident with *N* deaths.

For 1<N<50, the best fit (minimum square method) for a curve of type is $P=N^{b}$ and gave *b* = −1.010, as shown in Figure 9. A straight line with a slope of −1.010 was obtained by plotting the data on a log–log axis system. This indicates that the probability of an accident involving 10 or more deaths is 3.03 times greater than that of an accident involving 30 or more deaths.

### 3.4. Road Class of Accidents

The number of hazmat transportation accidents in China from 2013 to 2019 was obtained from the State Administration of Work Safety (SAWS). The type of road network data consists of national highways, provincial roads, county roads, township roads, and city roads. In this article, the road types are aggregated into four groups: group I (national highway), group II (provincial road), group III (county road and township road), and group IV (city road and others), as shown in Table 1 and Table 2 [23]. More than half of the accidents (60%) occurred on group I roads, followed by group IV (27.31%) and group III (7.36%) roads. In general, the national highway is the most connected roadway directed to hazmat factories and industries. Moreover, it also provides fast and convenient roads for hazmat transportation. Therefore, hazmat transportation accidents occurred most frequently on group I roads. In addition, group IV follows next, mainly because hazmat factories are generally built around the city. Therefore, in the process of transporting hazmat, they often travel to and from city roads. The situation of city roads is complicated, and it is easy to cause oil tank leakage due to improper operation of hazmat loading and unloading personnel. Serious accidents will probably occur, and the mortality rate is also high. Once the accident happens, the consequence is serious due to the residents living near the surrounding.

### 3.5. Causes of Accidents

According to the statistical results, hazmat transportation accidents can be roughly divided into six categories according to the causes, namely, weather factors, human factors, road factors, hazmat factors, vehicle factors, and other factors. There are still some unknown factors that are exceptions. The different number of accidents caused by various causes are shown in Table 1, which shows that 26.7% of cases were attributed to human factors, including driving distraction, fatigue driving, meeting cars, violation of traffic rules, improper overtaking, improper operation, improper turning, avoiding pedestrians, and the speed is too fast. The next most common set of causes consists of various types of hazmat factors (11.38%), such as tank and safety accessory failures. Each accident also consists of not only one reason, but several causes which may contribute effects. For instance, the cause of scratching and rolling of hazmat transport vehicles may be related to driver error, vehicle brake failure, and weather conditions.

### 3.6. Distribution of Accidents by Type and Road Classes

#### 3.6.1. Types of Accidents

The types of hazmat transportation accidents are classified into traffic accidents (such as collision, scraping, tipping, falling, etc., due to driver’s illegal operation, road conditions, vehicle conditions, or the environment) and non-traffic accidents (such as the failure of the vehicle itself or the leakage of the tank or valve directly leading to the explosion, fire, leakage, or spread). To be more specific, the types of accidents are divided into nine categories, as shown in Table 2. Rear-end, collision, collision with road facilities, rushing out of the road, scratches, and rollover are gathered as traffic accidents, accounting for 77.31%, and the rest are non-traffic accidents, in which the leakage is the most predominant type. Among the 2777 samples, rollover accidents (35.36%), rear-ends (22.58%), and collisions (14.87%) are the three most important types of accidents. The frequent occurrence of rollover accidents is mainly caused by the sloshing of the liquid in the transport vehicle or the liquid’s movement inside the tank, which will constantly change the weight of the tank. If there is not enough liquid in the internal tank, the truck may easily lose balance and thus roll over, especially during sudden evasive maneuvers or turning. There is a strong correlation between rollover and spillage, especially when the vehicle is in the entrance or exit ramp or a curved section. Accidents are most common in these locations. In addition, 3.82% of the accidents were caused by vehicle failures, for example, the doors or valves are not properly closed, or the brakes are on fire. Moreover, 6.73% of accidents are caused by the special nature of hazmat, such as the corrosivity. Therefore, strengthening the inspection and maintenance of vehicles and fuel tanks can effectively reduce such accidents.

#### 3.6.2. Road Classes of Accidents

First, more than half of the accidents (53.29%) occurred on the roads of group I, followed by group IV (33.27%) and group III (6.95%). It can be seen that China’s current national roads are regarded as the main transportation corridor and the black spots of hazmat transportation from the volume of trucks and the frequency of accidents. Second, many accidents occurred on the third and fourth groups of roads. This is mainly because the transportation of a large number of hazmat involves transportation to and from remote areas that are often served by city and county roads. In general, traffic crashes are the main type of accident in the different road groups.

### 3.7. Hazard Class for Materials Involved in the Accident

According to the template of the proposed regulations on the transport of hazmat, hazmat [30] is divided into nine categories. These are: explosives, gas, flammable liquid, flammable solids, oxidizing substances and organic peroxides, toxic substances and infectious substances, radioactive material, corrosive substances, and miscellaneous dangerous substances and articles. In Table 3, class 2 (17.79%), class 3 (56.07%), and class 8 (12.28%) accounted for a large amount of hazmat accidents. These distributions can be attributed to two main factors: First, the number of accidents in a particular hazard category will increase with their social needs and truck volume. Second, the reliability of vehicles and containers that transport flammable and explosive gases and corrosive materials decreased, as well as the reliability of materials that must be transported under high pressure and low temperature conditions, which also led to more and more accidents occurring.

### 3.8. Distribution of Hazard Consequences Involved in the Accident

During the transportation of hazmat, traffic accidents may occur due to road factors, environmental factors, and other factors, which may cause serious consequences such as leakage of hazmat, fire, explosion, and so on [31]. As shown in Table 4, leakage (80.34%) is the main type of consequence, in which 5.6% of fatal accidents occur. Although fire (8.32%) and explosions (2.34%) rarely happen during hazmat transportation, they usually result in more severe casualties. Fatal accidents account for 12.99% of fires and 29.2% of explosions. This is because the flammable liquid or gas has a large fluidity. If it is subjected to collision or leakage, it will easily cause an explosion, followed by a flammable gas accident. The flammable gas accident leakage may cause irreparable damage to the surrounding environment. Therefore, in the large-scale transportation of liquid and gas hazmat in China, to prevent accidents caused by liquid leakage of tank rupture, and even secondary accidents such as fire and explosions, strengthening the safe management of hazmat transportation should be focused on.

It is known that consequences of hazmat transport accidents will have an impact on the environment, such as on the road, air, water, and so on. From Table 3, it can be seen that class 3 and class 2 are the two most common kinds of hazmat, which means that liquid and gas are the main transportation materials. When they leaked, the pavement and road will be contaminated. Moreover, when the liquid or gas collision result in a fire or explosion, it will contaminate the air and soil.

Table 5 shows the proportion of the impact of hazmat accidents (e.g., road, air, soil, water, and so on). Road pollution is the highest at 62.8%, followed by air pollution at 20.8%. Some of the surroundings around the hazmat transport route are the river and farmland, and they would also become contaminated once the hazmat transportation accidents occur.

**Figure 10:**
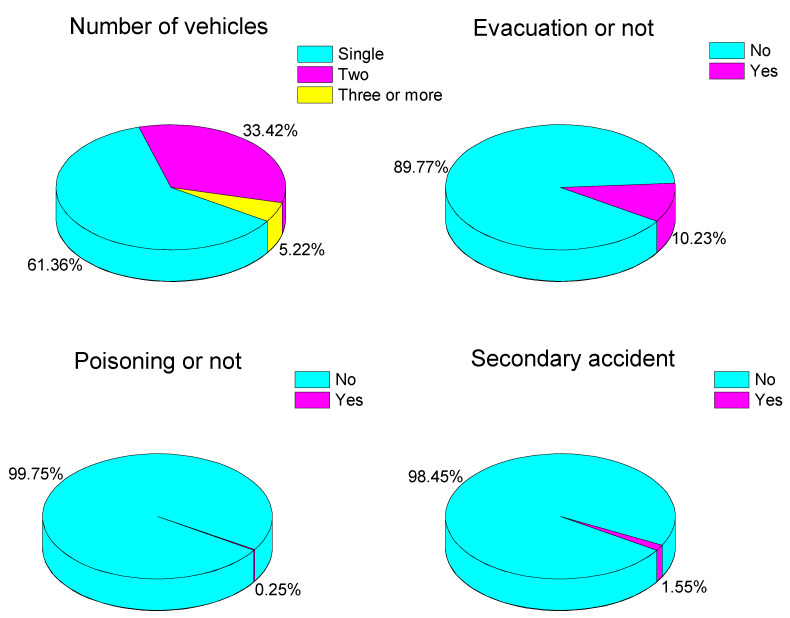
Distribution of Accidents by the number of vehicles, evacuations, poisonings, and secondary accidents.

In addition, from Figure 10, hazmat accidents involving a single vehicle accounted for 61.36% of total accidents, followed by two-vehicle accidents (33.42%). Hazmat accidents which have resulted in the evacuation of residents account for 10.23%, while nearly no one has benn poisoned (only 0.25%) and only 1.55% of accidents cause a secondary accident.

Single-vehicle accidents are generally caused by driver’s operation errors, vehicle failures, etc. Two-vehicle accidents generally occur on urban roads, mainly in the type of collision and scratching. Multi-vehicle accidents are generally caused by bad weather and slippery roads; thus, vehicle handling is reduced. When the transported material is toxic and the residential area is densely populated, the residents will be evacuated to protect their personal safety. A secondary accident generally occurs when a hazmat leaks or a leak caused by a traffic accident causes a fire or explosion.

## 4. Conclusions

This paper mainly analyzed the samples of 2777 hazmat historical transportation accidents from 2013 to 2019 in China from the aspects of time, geographical factors, road types, types of accidents, and causes and consequences of accidents. The degree of impact of accident factors on hazmat transportation accidents is obtained separately. In fact, in the past few decades, due to the increase in the number of tons per kilometer/year, the land transportation of hazardous materials has greatly increased, which is undoubtedly one of the reasons for this situation.

Of the total number of accidents found, August (10.05%) had the highest accident probability, followed by December (9.76%). This is probably due to relatively inclement weather during these two months, such as high temperature, rain, and snow. As a result, cooling and wetting in August and taking effective anti-skid and anti-freezing treatment of vehicles in December could be helpful.

Hazmat transportation accidents are more likely to occur from the periods of 6:00–9:00 a.m. and 9:00–12:00 a.m., while there is a significant downward trend of accidents from 18:00 to 24:00. This is probably due to the fatigue of the driver and daily traffic peak in the early morning. Therefore, it is necessary to make reasonable schedules to avoid the fatigued driving of drivers, which can effectively prevent accidents. Additionally, safety education and training are essential not only for the drivers and supercargoes of hazmat transportation but also for the public.

Furthermore, more than 34.35% of road accidents occurred in the East China areas, 14.87% in the northwest areas, 15.16% in the central inland areas, and only 3.75% in the northeast areas. Shandong (9.4%), Jiangsu (7.8%), and Zhejiang (6.7%) had the highest percentages of accidents. The conclusion is the same as He et al. [17]. The trends of the road class of hazmat accidents are also the same as in the conclusion of Shen et al. [32]. The probability of an accident involving 10 slightly injured people or more than 10 slightly injured people is 4.22 times greater than that of slightly injured involving 30 or more. In addition, the probability of an accident involving 10 or more deaths is 3.03 times greater than that of an accident involving 30 or more deaths.

More than half of the accidents (60%) occurred on the roads of group I, followed by group IV (27.31%) and group III (7.36%). On national roads, the rear-ending and rollover of vehicles are the most common accidents, meaning 33.42% of the total number of hazmat accidents occurred on national road. The single-rollover (61.36%) is the most typical accident on the group II road. On city roads, the cumulative proportion of accidents caused by tank leakage and vehicle failure is 10.55%. About 77% of the hazmat transportation accidents are caused by traffic accidents. Improving the safety level of driving is essential to avoid such accidents. Leakage during hazmat transportation caused by the leakage of tanks and accessories or termination of valves result in fires, explosions, etc. It is necessary to strengthen technical supervision of tank performance and reliability. The rollover of a single vehicle is the most important type of accident, accounting for 61.36% of the total number of accidents [33]. Human factors caused 26.74% of hazmat tanker transportation accidents. Improving the technical quality of hazmat transportation workers, especially the safety intentions of drivers and escorts, is an effective way to prevent hazmat transportation accidents [34].

These percentages show that class 2 (17.79%), class 3 (56.07%), and class 8 (12.28%) account for the majority of hazmat transportation accidents in China. Usually, leakage (80.34%) is the main type of consequence, while 8.32% of hazmat accidents result in is fire and 2.34% in explosion. Therefore, the impacts of hazmat accidents include road (62.8%) and air (20.8%) pollution. Moreover, hazmat accidents which have resulted in the evacuation of residents account for 10.23% of accidents, while there has been nearly no one poisoned, and only 0.25% accidents cause a secondary accident. This reveals that the evacuation and resettlement of residents is very important to hazmat transportation accidents.

In addition, consideration should be given to the existence of better and broader incident reporting practices, which may partially obscure these trends. However, the information collected seems to indicate that more and more accidents will occur in the next few years if security measures are not improved.

## Figures and Tables

**Table 1:** The number of accidents by cause of casualties and road classes.

Cause of Accidents	Group I			Group II		
	National Highway			Provincial Highway		
	Injury	Serious Injury	Mortality	Injury	Serious Injury	Mortality
Human factors	181	52	77	22	4	4
Weather	54	12	17	3	1	0
Road factors	24	13	9	2	0	2
Hazmat factors	63	24	25	2	0	0
Vehicle factors	30	8	14	4	2	2
Other	206	48	79	20	5	12
Unknown	129	24	60	12	2	3
**Cause of Accidents**	**Group III**			**Group IV**		
	**County Road and Township Road**			**City Road and Other**		
	**Injury**	**Serious Injury**	**Mortality**	**Injury**	**Serious Injury**	**Mortality**
Human factors	21	8	7	101	21	14
Weather	1	0	0	13	3	0
Road factors	1	0	3	32	12	3
Hazmat factors	27	4	4	39	19	11
Vehicle factors	1	0	2	24	10	2
Other	15	9	3	100	29	17
Unknown	15	7	13	53	11	9

**Table 2:** The number of accidents by type and road classes.

Type of Accidents	Group I	Group II	Group III	Group IV	Percentage
	National Highway	Provincial Highway	County Road and Township Road	City Road and Other	
Rear-end	454	24	15	134	22.58%
Collision	188	31	23	171	14.87%
Collision road facilities and rushing out of the road	41	3	5	29	2.81%
Scratch	22	7	3	15	1.69%
Vehicle breakdown	63	3	4	36	3.82%
Rollover	448	96	115	323	35.36%
Leakage	87	4	12	84	6.73%
Spontaneous combustion or Explosion	98	6	15	67	6.70%
Others	79	6	1	65	5.44%
Percentage	53.29%	6.48%	6.95%	33.27%	

**Table 3:** Distribution of Accidents by type of Hazmat.

Type of Hazmat	Class 1	Class 2	Class 3	Class 4	Class 5	Class 6	Class 7	Class 8	Class 9
Percentage of accidents	2.66%	17.79%	56.07%	1.51%	1.12%	1.33%	0.07%	12.28%	7.17%

**Table 4:** The number and percentage of accidents by consequences.

Consequences	Leakage	Fire	Explosion	Other
Percentage of accidents	80.34%	8.32%	2.34%	9.00%

**Table 5:** The impact of hazmat accidents.

Impact	Road	Air	Road and Air	No	Soil	Water	Other
Percentage	62.8%	20.8%	5.4%	4.1%	3.3%	1.5%	2.1%

## Data Availability

Data sharing is not applicable to this article.
